# A Single-Fed Multiband Antenna for WLAN and 5G Applications

**DOI:** 10.3390/s20216332

**Published:** 2020-11-06

**Authors:** Zakir Khan, Muhammad Hunain Memon, Saeed Ur Rahman, Muhammad Sajjad, Fujiang Lin, Liguo Sun

**Affiliations:** 1 Micro-/Nano-Electronic System Integration Center, University of Science and Technology of China, Hefei 230027, China; zakirkhan@mail.ustc.edu.cn (Z.K.); Mhm121@mail.ustc.edu.cn (M.H.M.); Linfj@ustc.edu.cn (F.L.); 2 College of Electronic and Information Engineering, Nanjing University of Aeronautics & Astronautic, Nanjing 211106, China; saeed@nuaa.edu.cn (S.U.R.); sajjadwazir@nuaa.edu.cn (M.S.)

**Keywords:** multibandoperation, slotted antenna, microwave, millimeter-wave band, WLAN, 5G

## Abstract

In this paper, a slotted conical patch connected to a small triangular patch multiband antenna for both microwave and millimeter-wave applications is presented. The designed antenna has three characteristics. The proposed antenna is a multiband, having a compact size of 0.35*λ*_0_ × 0.35*λ*_0_ × 0.004*λ*_0_ at its lowest operational frequency, i.e., 2.4 GHz, and more importantly, it can cover both the microwave and millimeter-wave bands with a single feeding. According to the −10 dB matching bandwidth, experimental results show that the antenna operates at (2.450–2.495) GHz, (5.0–6.3) GHz, and (23–28) GHz. The reduced size, simple design, and multiband large bandwidth are some of the advantages over the reported designs in the latest literature. Both simulated and experimental results show a good agreement, and the proposed antenna can be used for wireless local area network (WLAN) applications and fifth-generation (5G) wireless communication devices.

## 1. Introduction

A wireless local area network (WLAN) is a widely used network for short-range wireless communication applications. According to 802.11b/g and 802.11a standards, the bands used for WLAN are (2.400–2.484) GHz, (5.15–5.35) GHz, and (5.725–5.825) GHz [1]. Moreover, the need for high quality videos and other high data rate applications require wider bandwidth. Therefore, to fulfill the requirement of wider bandwidth, 28/38 GHz frequencies are seen as the most promising choices for fifth-generation (5G) technology [2].

Numerous researches have been exerted over the last few years pertaining to the evolution of existing standards of the wireless communication system to future 5G wireless communication standards, which is likely going to be implemented in the 2020s [3,4]. For that reason, more and more requirements have been made in the design of an antenna, in terms of size, multiband operation, and radiation pattern [5]. Many researchers are focusing on the advancement of an antenna system to operate in both current and future standards of wireless communication systems. Therefore, the acceptable way to be considered is the designing of a multiband antenna that can be integrated as a single element in many standards [6], such as WLAN, global positioning system (GPS), and other wireless communication applications.

For designing multiband antennas, different techniques were used previously to achieve multiband operating frequency standards [7,8,9,10,11,12,13,14,15,16,17]. The following study, of different multiband antennas covering the microwave band for WLAN applications, has been conducted in [1,7,8,9,10,11,12,13,14]. A defected ground structure (DGS) monopole antenna operating at triple frequencies for WLAN applications is presented in [6]. The radiating patch and ground of the antenna were etched on both sides of a printed-circuit board (PCB). The ground plane was modified by two equal-shaped slots on the right and left sides. Similarly, a multiband characteristic of the antenna in [9] was generated by a rectangular slot on the upper side of the antenna substrate loaded with differently shaped stubs on each side of the slot. In [10], a slotted monopole antenna, having a C-shaped patch introduced by a G-shaped parasitic strip and a partial ground plane, is used to obtain a larger bandwidth of 3.5 GHz at (3.92–7.52 GHz). Two elements of a multiple-input–multiple-output (MIMO) antenna etched with a different slot is reported in [13]. Similarly, a triple-band antenna for 2.4, 5.2, and 5.8 GHz applications in [1] and a dual-band antenna operating at 2.4 GHz and 5.2 GHz in [14] are presented. Meandering slots etched in the patch and a slotted ground DGS is used, respectively, to obtain the triple and dual-band characteristics. In [15], a 28-GHz mm-wave antenna of size 30 mm *×* 20 mm for 5G is reported, which is the combination of a waveguide aperture and several microstrip patches. Further, the study of antennas covering both microwave and mm-wave bands simultaneously were performed in [16,17]. In [16], a multi-layer antenna system having a dual-element MIMO on the top layer operating in the microwave band, and an antenna array at the bottom layer for the 5G band, is presented. A multiband antenna operating in both microwave and mm-wave is introduced in [17], which consists of a monopole antenna operating at 2.4/5.5 GHz and a rectangular patch covering the mm-wave 5G band. The comparison of the proposed work with the available designs of [7,8,9,10,11,12,13,14,15,16,17] in terms of bandwidth, multiband operation, substrate availability, design complexity, the number of layers, and feeding used is shown in Table 1. It was observed from the comparison table that the available designs have large size, complex geometry, multi-ports, and they can only cover the microwave band, or only mm-wave band, but cannot cover both bands with one feeding. Thus, the challenging part of this work is to design an antenna that can cover both the microwave band and mm-wave band with a single feeding and a compact size.

To solve these problems (of large size and complex geometry), a compact, multiband antenna covering the microwave band and the mm-wave band is proposed. Rogers RT/Duroid 5880, a widely available and inexpensive substrate was used to design the proposed antenna. We modified the radiating patch by truncating the corners of the two rectangles to form a cone and a triangle. We etched different slots in the conical patch, which increased the electrical length of the antenna and made it more compact. Additionally, corners of the ground plane were truncated and cut by different slots to form a DGS, unlike the conventional solid ground plane. The mentioned design techniques were applied to make the antenna resonate at about (2.4, 5.2, 5.8) GHz and (28 GHz). To endorse the concept and validate the simulated results, a prototype is fabricated and results are measured. The simulated and measured results suggest that the designed antenna is the best candidate for various wireless communication applications in terms of multiband operations, compactness, large bandwidth, ease of design, and low cost. In Section 3, an explanation of a parametric study has been discussed to properly select optimized dimensions of the proposed design and achieve good results of multiband. In Section 4, different types of results were discussed followed by the comparison and conclusion of the paper in Section 5 and Section 6.

## 2. Geometry of the Antenna

**Figure 1:**
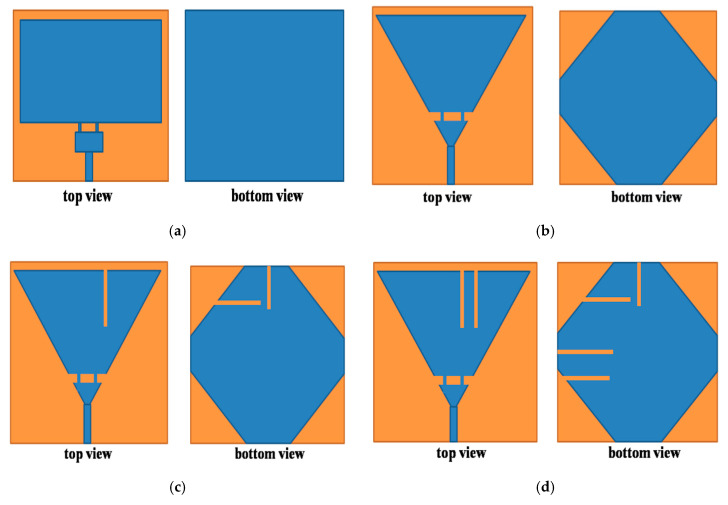

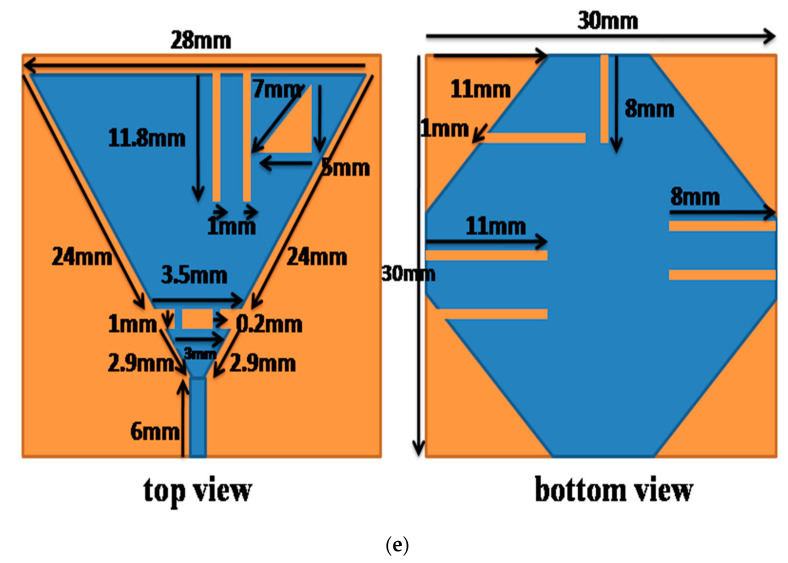
Evolution of proposed antenna front and back view (**a**)Antenna1, (**b**) Antenna2, (**c**) Antenna3, (**d**) Antenna4, and (**e**) proposed antenna.

The detailed geometry of the proposed antenna will be discussed in this section. The three-dimensional (3D) electromagnetic wave solver, computer simulation technology (CST) microwave studio [18] was used for numerically investigating and optimizing the configuration of the proposed designed antenna. The front and back view of the antenna is depicted in Figure 1e. From figure, the blue color is the copper used at the top and bottom layer and the brown color is the substrate. Rogers RT5880 (*ε_r_*= 2.2, tan *δ =* 0.0009) is used as a substrate to design the antenna. The overall dimension is 30 *×* 30 *×* 0.508 mm^3^. The final geometry consists of a slotted conical patch connected to a small triangle by narrow lines. The conical patch covers the WLAN band and the triangular patch covers the 5G band. Two meandering slots and a triangular slot were etched on a conical patch. Feeding is given through a 50 Ω microstrip line. A defected ground plane etched with six slots and truncated corners are used at the bottom layer of the antenna.

**Figure 2:**
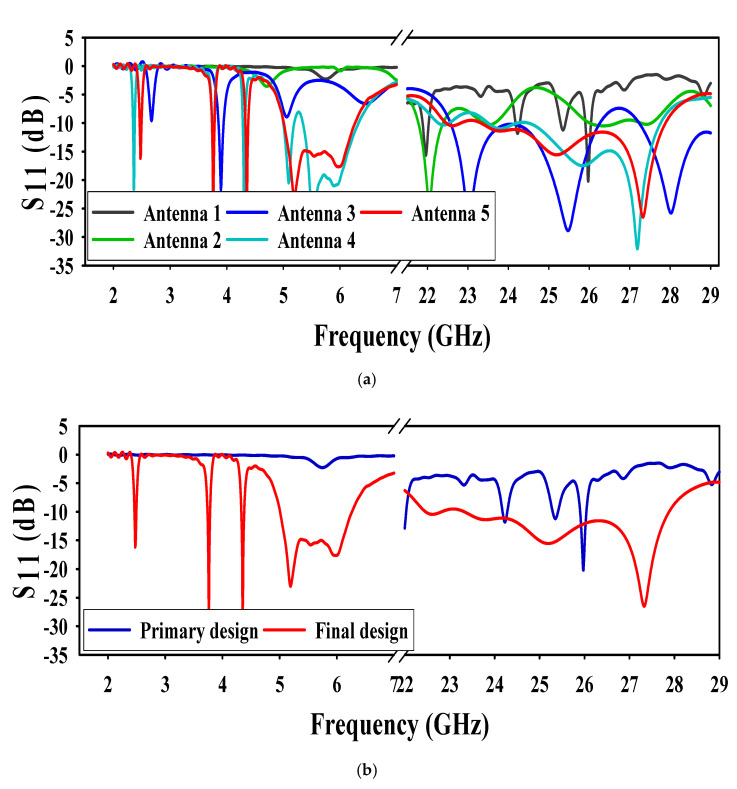
Simulated S_11_ (**a**) antenna 1 to 5, (**b**) primary and final design.

The final geometry of the designed antenna was obtained by different modifications in two rectangular patches antenna (Antenna1) shown in Figure 1a. The Antenna1, patch dimensions such as width and length were obtained by Equations (1) and (2) [19]. From Figure 2a,b the Antenna1 is resonating at (24.32–24.54) GHz and (25.5–25.7) GHz. It also gives resonance at (5.3–6.0) GHz but that’s not below −10 dB. We used corners truncation and meandering slits for compactness and multiband operation [6,9]. These two techniques lower the frequency of operation of the antenna by increasing the electrical length that results in the compactness of the antenna [6,9,20,21]. The ground plane used at the bottom layer is defected with different meandering slots and truncated corners to get higher bandwidth in both operating bands of the antenna. More details of truncating the corners of the square patches to form a conical and triangular patch are also discussed in Section 3.1 of this article. The next stage (Antenna2) in Figure 1b had its corners truncated from rectangular patches and the ground plane to form a cone-like and a small triangle. From Figure 2a, the resonating frequency of Antenna2 is lowered to (21.80–22.42) GHz and (26.2–27.6) GHz. Moreover, resonating at 4.7 and 7.0 GHz, but the resonance was not below −10 dB. In the next stage, Antenna3, a slot of optimized value in the conical patch and two slots in the truncated ground were etched as shown in Figure 1c. From Figure 2a, the higher resonating frequency turns into a broad band, i.e., (22–26) GHz. While at the lower band, the antenna resonates at 3.9 GHz, which is below −10 dB. It also resonates at 2.60 and 5.05 GHz, but that is above −10 dB. Moving to the next stage, Antenna4, one more slot in the patch and two more slots in the ground plane were etched, shown in Figure 1d. The antenna resonates at 2.3 GHz and (5.0–6.3) GHz, but there is a mismatch at 5.2 GHz. Thus, further improvement is needed. The final stage is to etch a triangular slot in the patch along with two more slots in the ground plane, shown in Figure 1e. From Figure 2a,b it can be seen that the antenna is exactly resonating at (2.46–2.49) GHz, (5–6.3) GHz, and (23–28) GHz.

(1) $$W=\frac{\left(2N+1\right)}{\sqrt{\left({Ɛ}_{r}+0.5\right)}}\times \;\frac{\lambda o}{2}\;$$

(2) $$L=\frac{\left(2N+1\right)}{\sqrt{{Ɛ}_{eff}}}\times \;\frac{\lambda o}{2}-2\Delta L$$

Ɛ_r_ = dielectric constant, λ*_o_* = free space wavelength.

∆L is the effective length and can be found by the Equation (3)

(3) $$\frac{\;\Delta L}{h}=0.412\left[\frac{\varepsilon_{eff}+0.3}{\varepsilon_{eff}-0.258\;}\right]\left[\frac{\frac{w}{h}+0.264}{\frac{w}{h}+0.813}\right]$$

where ℇ*_eff_* = effective relative permittivity of the substrate

(4) $$\varepsilon_{eff}=\frac{\varepsilon_{r}+1}{2}+\frac{\varepsilon_{r}+1}{2}\sqrt{\left(1+12h/2w\right)}$$

## 3. Parametric Study

To understand the impact of various parameters on different results and to achieve the best optimized dimensions of the final design, a parametric analysis has been done on different parameters of the antenna. All other parameters were kept at their final value during the parametric study.

### 3.1. Effect of Truncating Corners of the Patch

**Figure 3:**
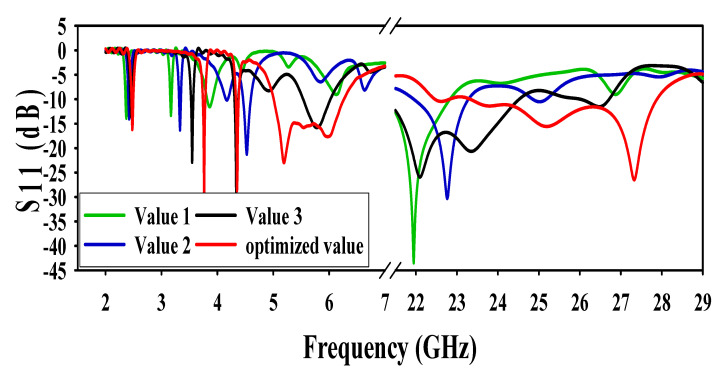
Effect of truncating the corners of patches.

The corners of the rectangular patches shown in Figure 3 were truncated at three different values to form a conical and triangular shape patch. A visible effect at both operating frequency bands i.e., microwave and mm-wave band, was observed. At first value, the antenna is only resonating at 2.4 GHz in the microwave band while at (21 GHz–22.5 GHz) in the mm-wave band. At the second value, the antenna resonating frequencies are 2.4 GHz, (4 GHz–5 GHz), and (22 GHz–23.5 GHz). At the third value, the antenna gives resonance at 2.4 GHz, 5.8 GHz, and (21 GHz–24.8 GHz). The effect of different values along with the optimized value results are shown in Figure 3.

### 3.2. Effect of Truncating Corners of the Ground

**Figure 4:**
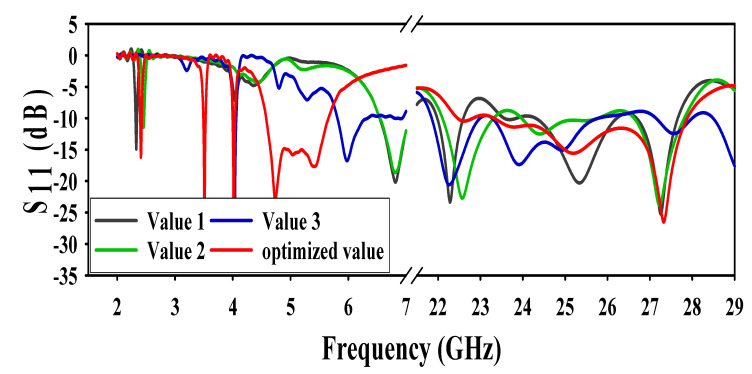
Effect of truncating corners of the ground.

The DGS also has a very high impact on both microwave and mm-wave bands of operation of the antenna. Two types of techniques were used in this paper for defected ground. The first method was a truncation of the ground at three different values at all corners. At first value, the antenna resonated at 2.3 GHz, whereas the other resonating frequency shifted to 6.5 GHz. Moreover, there was a mismatch at (22.5–24.3) GHz. At the second value, the resonance frequency shifted to 2.4 GHz and 6.4 GHz, and a mismatch at (25.5–26.5) GHz. Finally, when the value increased from its optimized value, the resonance frequency moved to 4 GHz and 5.8 GHz. Whereas, at mm-wave, there was mismatching at (25.7–27.1) GHz. All of the results (of truncating the corners of the ground at different values along with its optimized values) are shown in Figure 4.

### 3.3. Effect of Different Values of Slots in the Ground

**Figure 5:**
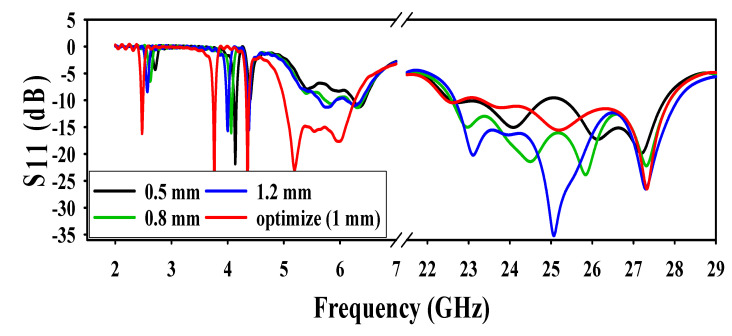
Effect of different values of slots in the ground.

The second technique used for DGS was to etch different values of meandering slots at the bottom layer. Widths of the ground slots were varied at different values to analyze its performance at all the operating frequencies. Each slot width decreased to 0.5 mm from its optimized value (1 mm) and it was observed (from the result shown in Figure 5) that the antenna was not resonating at 2.4, 5.2, and 5.8 GHz. Again, when width of the slots increased from 0.5 to 0.8 mm, the resonance was above −10 dB. Finally, when the width of each slot increased to 1.2 mm, the antenna resonated only at 5.8 GHz, with the maximum resonance of −12 dB. There is no clear effect on the mm-wave operation band apart from a little mismatch at 0.5 mm on (21–25) GHz. The effect of variation of slots in the ground layer is shown in Figure 5.

### 3.4. Effect of Distance between Two Patches

**Figure 6:**
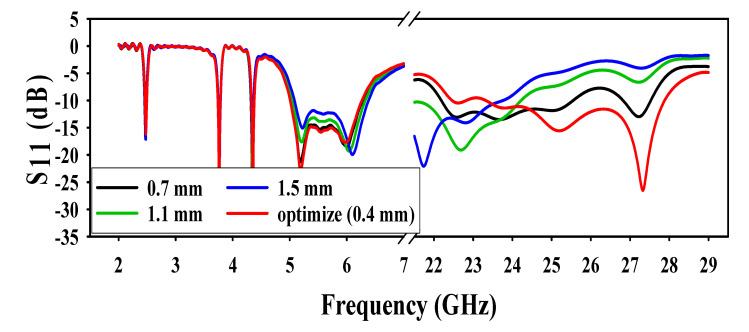
Effect of distance between patches.

The antenna performance was analyzed by different values of spacing between a triangle and a conical patch. As illustrated in Figure 6, by increasing the distance between the patches, the resonance at the mm-wave band deteriorated with every variation, and had almost no effect on the lower frequencies. Initially, the distance between two patches was kept at 0.7 mm and a mismatch was observed at (25.5–27.8) GHz. When the distance further increased to 1.1 mm, the resonating frequency emanated to (22–24.2) GHz. Finally, when the distance was kept at 1.5 mm, then the resonance came further down to (21–23.5) GHz.

### 3.5. Effect of Different Values of Slots in the Patch

**Figure 7:**
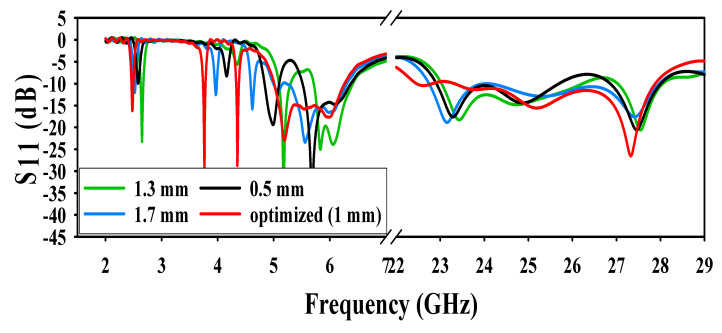
Effect of different values of slots in the patch.

The conical patch slots width were varied at three different values and the results were analyzed. In the first step, the widths of the slots were kept 0.5 mm, and it was noted that the antenna was resonating at 5.2 GHz and 5.8 GHz, and the resonance frequency of 2.4 GHz shifted to 2.6 GHz. When widths of the slots increased to 1.3 mm, it was observed that the antenna was only resonating at 5.8 GHz, whereas there was no resonance at 2.4 GHz and 5.2 GHz. Finally, when the width of the slots further increased to 1.7 mm, again, the antenna only resonated at 5.8 GHz, and there was no resonance at 2.4 GHz and 5.2 GHz. From Figure 7, there is no effect of patch slot variation at the mm-wave operating frequency.

## 4. Simulated and Measured Results Discussion

**Figure 8:**
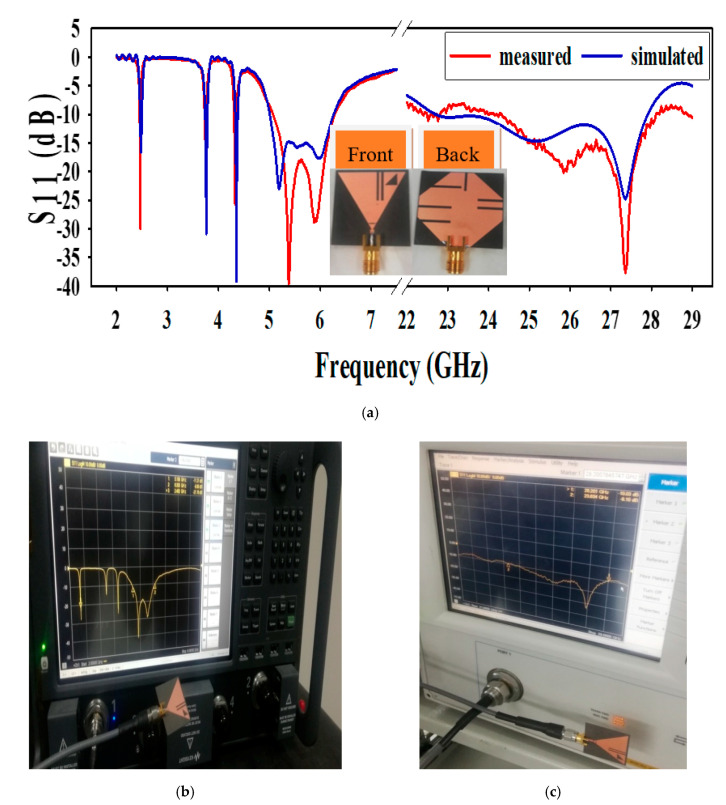
(**a**) Simulated and measured S-parameter, (**b**) measurement setup 2–9 GHz, and (**c**) measurement setup 21 to 29 GHz.

The prototype antenna, shown in Figure 8a, is fabricated and measured to confirm simulated results. Different results of the proposed antenna, such as reflection coefficient, radiation pattern, current density, and antenna gain will be discussed in the subsections below.

### 4.1. Reflection Coefficient

The proposed antenna simulated along with measured S_11_ results are depicted in Figure 8a. The S_11_ result from 2 to 7 GHz was measured by the subminiature version A (SMA)-1 connector (D550B51H01-118) and the S_11_ result from 23GHz to 28 GHz was measured by SMA-2 2.92 (D360B50H01-118). The S_11_ value is below −10 dB at all frequencies of operation. The antenna operates at different frequency bands, i.e., in microwave band at (2.45–2.495) GHz, (5.0–6.3) GHz and in mm-wave band at (23–28) GHz. The demonstrated measurement setup for S_11_ is shown in Figure 8b,c, measuring the microwave and mm-wave band respectively. In Figure 8a–c, a very good agreement can be seen between the results of simulated and measurement. However, the slight dissimilarity between the two results, especially at the mm-wave band of operation, can be noticed, and it could be possible because of the practical factors, which include SMA connector loss and hand soldering of the SMA-D360B50H01-118 to the antenna.

### 4.2. Current Density

**Figure 9:**
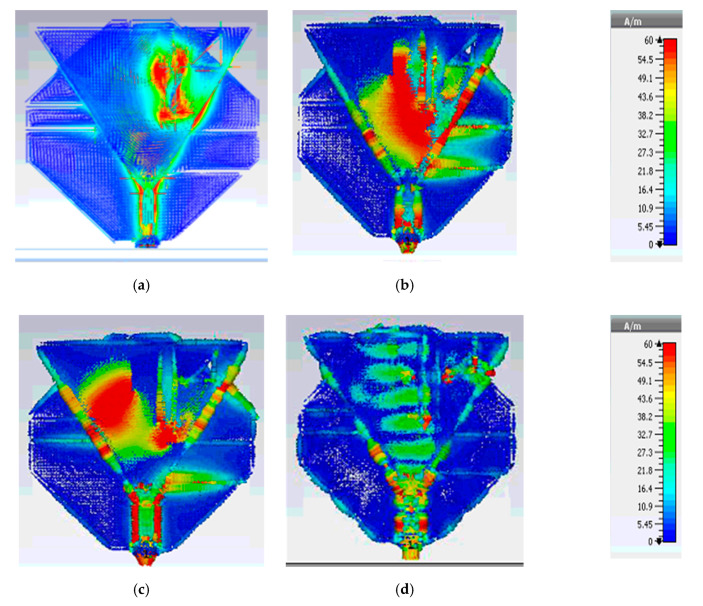
Surface current density (**a**) 2.4 GHz, (**b**) 5.2 GHz, (**c**) 5.8 GHz, and (**d**) 27.5 GHz.

To understand further explanation of the multiband operation, the surface current distribution of the designed antenna was analyzed at 2.4, 5.2, 5.8, and 28 GHz. As shown in Figure 9a–c, the maximum current density is along the different parts of the conical shape patch at 2.4 GHz, 5.2 GHz, and 5.8 GHz. Whereas in Figure 9d, it can be realized that the maximum current strength of the antenna is mainly associated with the smaller triangle at 27.5 GHz.

### 4.3. Radiation Pattern

**Figure 10:**
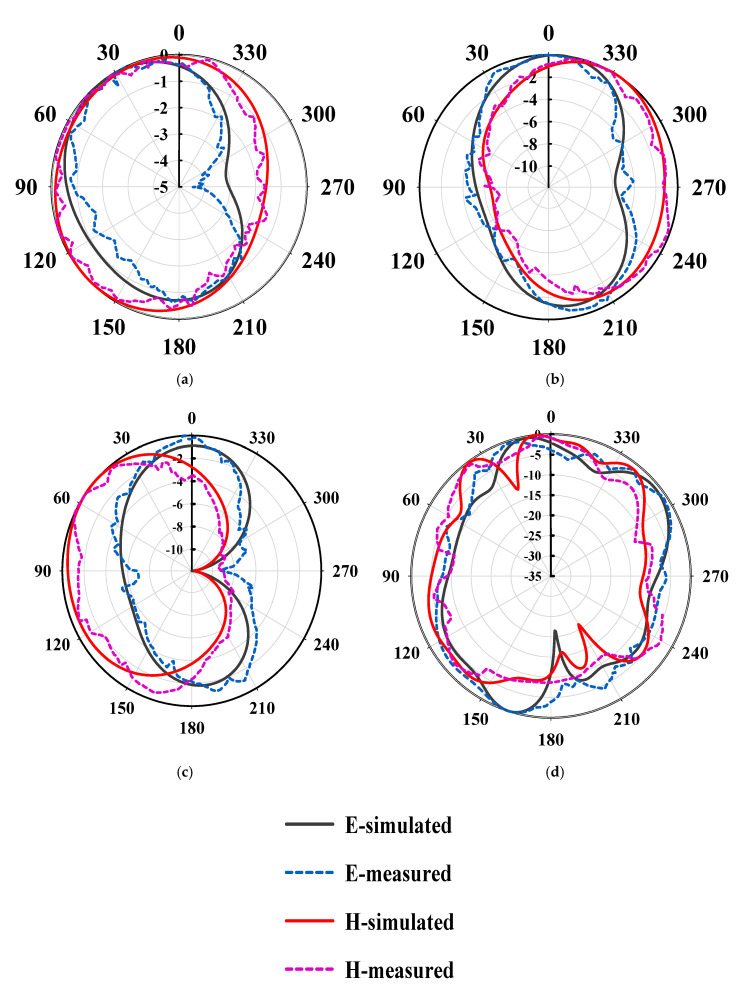
Radiation pattern in E-plane and H-plane at (**a**) 2.4 GHz, (**b**) 5.2 GHz, (**c**) 5.8 GHz, and (**d**) 26.5 GHz.

The far-field radiation pattern and gain were measured at 2.4 GHz, 5.2 GHz, 5.8 GHz, and 26.5 GHz. Both the E-plane and H-plane radiation patterns were given in Figure 10a–d. The radiation pattern in E-plane at 2.4 GHz, 5.2 GHz, and 5.8 GHz is nearly a dumbbell shape, whereas it is nearly omnidirectional in H-plane, which makes it suitable for multiple wireless systems. Similarly, the E-plane and H-plane radiation pattern at 26.5 GHz nearly has a directional pattern, as shown in Figure 10c. The measured radiation pattern results have a good agreement with the simulated results. However, again, a minor discrepancy can be noticed, and it could be possible as a result of the hand soldering of the 2.92 mm SMA D360B50H01-118 connector and measurement errors.

### 4.4. Antenna Gain

**Figure 11:**
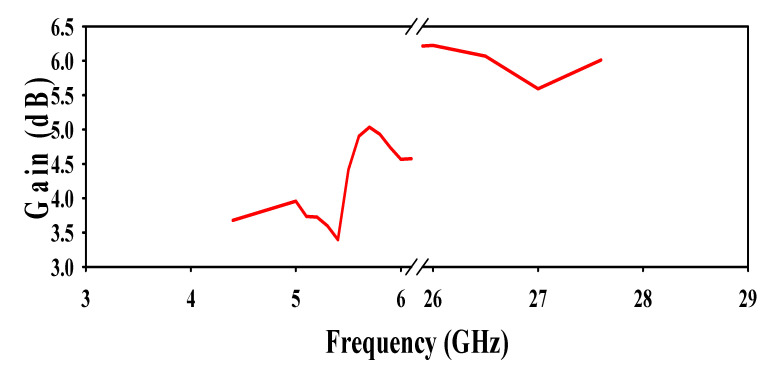
Proposed antenna gain over frequency.

**Figure 12:**
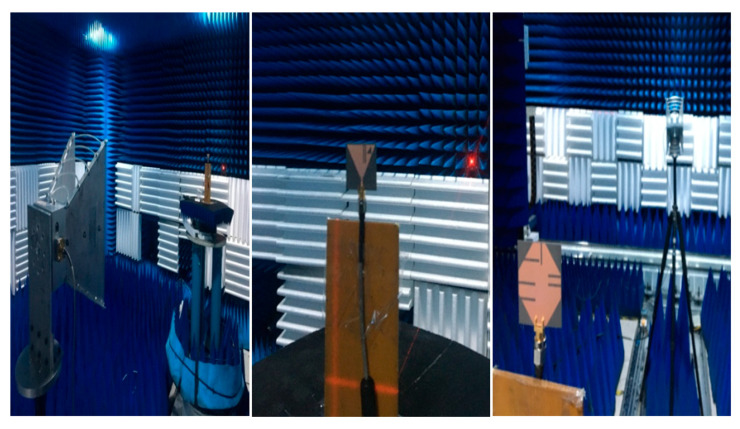
Measurement setup for radiation pattern.

The antenna gain was calculated using an anechoic chamber at different frequencies of operation in both microwave and the mm-wave bands are depicted in Figure 11. A horn antenna was used as a reference antenna and the measurement setup in the anechoic chamber can be seen in Figure 12. From Figure 11, it can be seen that the antenna gives a maximum gain of 3.55 dB at 5.2 GHz, 4.72 dB at 5.8 GHz, and 5.85 dB at 26.5 GHz, respectively.

## 5. Comparison

The comparison of the proposed design with other state-of-the-art designs is presented in Table 1. It can be seen in Table 1 that the antenna designs in the literature can only operate in microwave band for WLAN applications or in mm-wave band for 5G applications while the proposed designed antenna in this paper can operate in both microwave and mm-wave bands. The proposed antenna can be useful for two different communication technologies. Moreover, the proposed design can cover a large bandwidth as compared to the available designs. Further, the proposed antenna designed in this paper gives better performance in terms of multiband operation, light weight, low profile, low fabrication cost, simple geometry, and compactness.

## 6. Conclusions

In this paper, a DGS slotted double patch antenna, having a compact size, single feeding, simple design, and multiband characteristics, was designed and measured. The designed multiband antenna consists of (2.4, 5.2, and 5.8) GHz slotted conical patch antenna and 28 GHz triangular patch antenna. Good results were achieved in both the microwave band and mm-wave band. High-quality results compared to the latest literature were obtained by optimizing different parameters of the antenna. Measured results confirmed the proposed antenna is a suitable candidate for WLAN (5.0–6.3) GHz and 5G (23–28) GHz applications.

## Figures and Tables

**Table 1:** Comparison of different multiband designs.

Ref.	Dim (mm^2^)	Frequency (GHz)	Remarks
[6]	20 × 30	(2.14–2.52) (2.8–3.7) (5.15–6.02)	Cover only microwave band.
[9]	56 × 44	(1.57–1.66), (2.40–2.54), (3.27–3.97), (5.17–5.93)	Cover only microwave band.
[10]	32 × 30	(3.92–7.52)	Cannot cover 2.4 and the Mm-wave band.
[11]	24 × 16	(5.15–5.35), (5.72–5.82)	Cannot cover 2.4 and the Mm-wave band.
[15]	20 × 30	(26–29)	Only the mm-wave band.
[16]	60 × 100	(1.870–2.530) (26–28)	Complex geometry, Dual port and cannot cover 5.2, 5.8 GHz.
[17]	45 × 40	(2.16–2.53), (4.58–5.80), (26.8–30.0).	Large size, Complex geometry.
This work	30 × 30	(2.46–2.49), (5.0–6.3), (23–28)	Covers microwave, mm-wave band, with simple geometry and compact size.
